# Shared and distinct neural correlates of first and second language morphological processing in bilingual brain

**DOI:** 10.1038/s41539-023-00184-9

**Published:** 2023-09-04

**Authors:** Fei Gao, Lin Hua, Paulo Armada-da-Silva, Juan Zhang, Defeng Li, Zhiyi Chen, Chengwen Wang, Meng Du, Zhen Yuan

**Affiliations:** 1 Centre for Cognitive and Brain Sciences, University of Macau, Macau SAR, China; 2 Institute of Modern Languages and Linguistics, Fudan University, Shanghai, China; 3 Faculty of Health Sciences, University of Macau, Macau SAR, China; 4 Faculty of Education, University of Macau, Macau SAR, China; 5 Faculty of Arts and Humanities, University of Macau, Macau SAR, China; 6 The Affiliated Changsha Central Hospital, Hengyang Medical School, University of South China, Changsha, China; 7 Institute of Medical Imaging, Hengyang Medical School, University of South China, Hengyang, China; 8 The Seven Affiliated Hospital, Hengyang Medical School, University of South China, Changsha, China; 9 School of International Cultural Exchange, University of Finance and Economics, Central Beijing, China

**Keywords:** Language, Human behaviour

## Abstract

While morphology constitutes a crucial component of the human language system, the neural bases of morphological processing in the human brain remains to be elucidated. The current study aims at exploring the extent to which the second language (L2) morphological processing would resemble or differ from that of their first language (L1) in adult Chinese-English bilinguals. Bilingual participants were asked to complete a morphological priming lexical decision task drawing on derivational morphology, which is present for both Chinese and English, when their electrophysiological and optical responses were recorded concurrently. Functional near-infrared spectroscopy (fNIRS) revealed a neural dissociation between morphological and semantic priming effects in the left fronto-temporal network, while L1 Chinese engaged enhanced activation in the left prefrontal cortex for morphological parsing relative to L2 English. In the early stage of lexical processing, cross-language morphological processing manifested a difference in degree, not in kind, as revealed by the early left anterior negativity (ELAN) effect. In addition, L1 and L2 shared both early and late structural parsing processes (P250 and 300 ~ 500 ms negativity, respectively). Therefore, the current results support a unified competition model for bilingual development, where bilinguals would primarily employ L1 neural resources for L2 morphological representation and processing.

## Introduction

As a crucial component of the human language system, morphology implicates the formation of words and their inter-connections within a language. With respect to word formation, three strategies were generally employed across languages, that is, inflection, derivation, and compounding. Morphological typology classified world languages in light of these word formation methods e.g., ref. ^1^. For instance, Chinese is a typical isolating language which relies heavily on compounding (more than 70%) for word formation instead of inflections. As such, Chinese is also called a morpho-syllabic language, where one character/syllable is linked to one specific morpheme in the most cases^2^. By contrast, Latin constitutes an instance of inflectional language using extensive inflections. In particular, modern English is regarded as a weakly inflectional language with limited word form changes marking number, tense, among others, although it also belongs to the Indo-European language family as Latin. Despite the linguistic typology, morphology and word structure information generally implicate an important processing stage and component in both language comprehension and production processes^3,4^. Extensive studies shed light upon the notion that the competence in morphological processing could impact complex word comprehension, early literacy development, and reading in a second language e.g., refs. ^5–8^. So far, however, the neurobiological basis of morphological processing in the human mind and its brain bases across languages and populations is still poorly understood.

Drawing on electrophysiological and neuro-imaging techniques, extant studies arguably elucidated that morphological processing constitutes an integral part in the human mental lexicon, which is dissociable from semantics^9,10^. Bölte, et al.^9^ tracked the brain potentials to German derived adjectives with altered morphological manipulations by using event-related potentials (ERPs) and reported a left anterior negativity (LAN) effect peaking around 450 ~ 500 ms after word onset. LAN has been recognized as the indicator of sensitivity to (morpho)syntactic errors^11,12^ in existing studies. The authors therefore interpreted the observed LAN as an index of structural difficulty resolving and morphological parsing. Likewise, Gao, et al.^13^ identified N400 and LAN effects associated with semantic and morphological constraints, respectively, when Chinese native speakers were reading real compound words, morphologically legal, and illegal nonwords. As such, morphological processing might implicate a late, controlled, and top-down process. In contrast, earlier ERP components, such as P/N250, were also found to be associated with an automatic and form-based morphological decomposition in both English and Chinese word reading^14–16^. Yet, this early and rapid component is present only in a masked morphological priming paradigm with a short stimulus-onset asynchrony (SOA range: 50 ~ 57 ms in the abovementioned studies).

Meanwhile, results from magnetoencephalography (MEG) and functional magnetic resonance imaging (fMRI) suggested an important role of the left frontal and temporal cortices in morphological processing across languages^17–24^. Within this network, the left frontal gyrus (LFG) tends to be a language-general region for morphology, sometimes accompanied with the basal ganglia and the cerebellum^25,26^. A line of Chinese studies also suggested that the left temporal regions might be crucial in Chinese morphological tasks, which might correspond to the morpho-syllabic salience of Chinese language as compared to alphabetical languages. For instance, a recent fMRI study^27^ asked Chinese adults to complete morphological and phonological judgment tasks, respectively. Their results revealed that morphological tasks elicited robust activations in the superior temporal gyrus (STG) and middle temporal gyrus (MTG), relative to phonological tasks. In addition, another MEG study^28^ identified the altered activations in the left posterior temporal cortex, which was modulated by the morphological complexity in reading Chinese two-syllabic words. Note that as the aforementioned studies involved various morphological structures (inflections, derivations, and compounds), their findings on language-general and/or specific neural basis for morphological processing should be interpreted with caution.

So far, however, there has been little discussion about the neural correlates of morphological processing in bilingual brains. As bilingualism becomes increasingly pervasive in the globalization context, insights into bilingual brain would be informative for biliteracy development and pedagogy. It is thus crucial to investigate whether or not bilinguals would employ the same morphological processing strategies for first (L1) and second languages (L2). Even though a number of studies compared the morphological representation and processing patterns of bilingual individuals’ L2 with that of native speakers speaking the same language e.g., refs. ^5,29–31^, the relationship between L1 and L2 within bilingual brain has not been well established. This inadequacy in L1/L2 comparisons within bilingual morphological processing could be largely attributed to the confounding effect from typological difference between L1 and L2. Methods have been used to somewhat mitigate this confound in contrasting L1 and L2 among bilingual research, which include recruiting highly proficient bilinguals and using shared language structures between L1 and L2. For instance, Lehtonen, et al.^30^ observed language-specific brain patterns for L1 Finnish and L2 Swedish when highly-proficient Finnish-Swedish early bilinguals completed a lexical decision task with inflected and mono-morphemic nouns during fMRI scans. Specifically, L1 morphological processing was linked to left IFG and posterior temporal area (PTA), which were however not observed in L2 Swedish. This pattern thus suggested distinct processing strategies for bilingual morphology. In contrast, an increasing body of literature tended to hold that neural mechanisms underlying L2 morpho-syntactic processing are transferred from L1 system when L1 and L2 share the same structures see the review by ref. ^32^. This notion is primarily in line with the unified competition model^33^, where the processing of L1-L2-overlapped language structures would employ common cognitive resources and strategies from L1. For example, bilingual morpho-syntactic processing engages similar fronto-temporal network for L1 and L2 e.g., refs. ^34,35^. In addition to shared neural resources, there are also differences between L1 and L2 within bilinguals, generally resulting from L2 proficiency, age of acquisition (AoA), and linguistic variations. With respects to morphological processing, the extent to which bilingual L2 resembles or differs from their L1 brain patterns still remains unclear.

Therefore, the current study aims at investigating the shared and distinct neural correlates of L1 and L2 morphological processing of shared structures among Chinese-English bilinguals. A recent study^36^ contrasted the neural corelates of morphological processing among Chinese-English bilingual children with English monolinguals (6 ~ 13 years) by using auditory morphological task. Interestingly, compared to English monolinguals, bilingual children manifested enhanced brain activation in the MTG for English task, which is closely associated with lexico-semantic processing and second language literacy. Meanwhile, L2 English morphological processing involved greater IFG activation relative to L1 Chinese within bilingual individuals. The results therefore suggested both language-general and language-specific neural resources for L1 and L2 morphological engagement. In particular, the authors interpreted the alternations in the IFG between L1 and L2 as a language-specific indicator, which revealed bilingual children’s sensitivity to differing morphological constraints of Chinese and English. However, they employed distinct morphological structures (i.e., English derivation and Chinese compound), which might contaminate the bilingual transfer effect as they claimed. Moreover, morphological awareness elicited from auditory tasks might not fully capture the morphological salience, which relies largely on meaning-to-print association. Therefore, the objective of the current study is to further examine the shared and distinct neural corelates of L1 and L2 morphological processing in bilingual brain by using a visual morphological priming paradigm and a shared morphology. Both electroencephalogram (EEG) and functional near infrared spectroscopy (fNIRS) would be recorded simultaneously to capture the temporal-spatial changes in the brain when adult Chinese-English bilinguals were performing morphological tasks. By using a derivational morphology, which exists in both Chinese and English, we hypothesized the observed temporal-spatial characteristics would denote the processing strategies of L1 and L2, instead of linguistic difference.

## Results

### Behavioral results

**Fig. 1:**
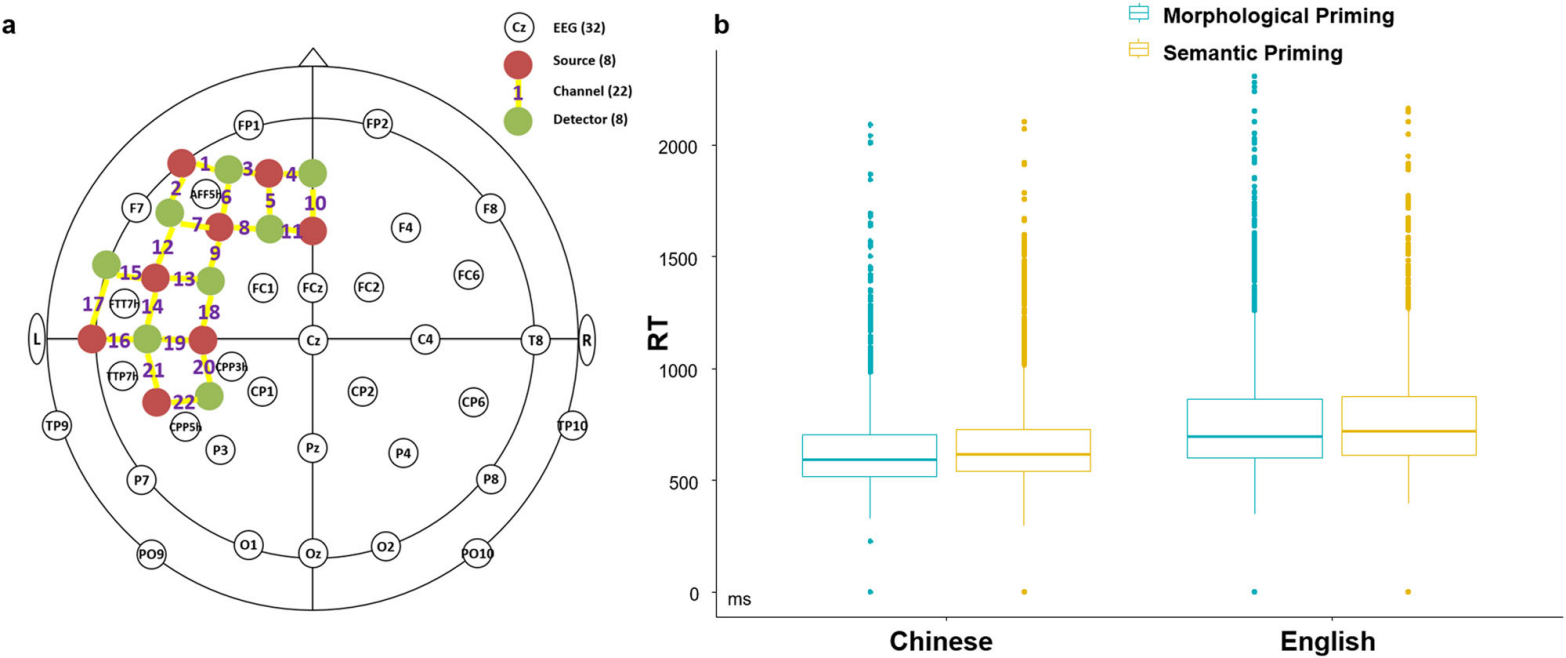
EEG-fNIRS layout and behavioral results. **a** There were 32 EEG electrodes (white) and 22 fNIRS channels (yellow) consisting of 8 LED sources (red) and 8 detectors (green). **b** Reaction time (RT) results showed significant main effects of language and priming type, as well as a marginal significant interaction. The middle line of each box denotes the data median, while the upper/lower bounds of the box show the interquartile range. The whiskers denote the bottom 25% and top 25% of the data range. Discrete dots represent outliers.

Reaction time (RT) and accuracy rate (ACC) were calculated from each participant across all conditions. The averaged ACC across all participants was 94.90%, suggesting that all participants were well engaged in the experiment. Responses which deviated more than 3 standard derivations (SD) from the mean RT were removed from further analysis, which took up less than 1.9% of all test items. Note that the discrimination of people vs. non-people targets was to avoid participants’ response strategy in a block design, whose corresponding responses were not treated as an independent factor. The cleaned data were submitted to a linear mixed-effect model by using lme4 package and ANOVA function in RStudio^37,38^. Specifically, language and priming type were treated as fixed factors, while people-relatedness, normalized word length of target words, semantic distance between primes and targets, participants’ age of learning English, and LexTALE scores served as covariates. Meanwhile, participant was included as random factor. RT results (Fig. 1b) indicated a significant main effect of language (estimate = 126.029, *t* = 3.721*, p* < 0.001, 95% CI = [0.06, 1.00]), such that Chinese items were recognized faster than English items. There was a significant priming type effect (estimate = 20.666, *t* = 2.864, *p* < 0.01, 95% CI = [0.00, 1.00]), where items of morphological priming were recognized faster than that of semantic priming. The interaction between language and priming type reached a marginal significance (estimate = −18.977, *t* = −1.841, *p* = 0.066, 95% CI = [0.00, 1.00]), whose following analysis showed that Chinese morphological priming condition was associated with faster responses than Chinese semantic priming (*p* < 0.01). In addition, ACC data showed a significant language effect (estimate = 0.578, *z* = 3.535, *p* < 0.001), such that Chinese items were recognized more accurately than English ones. However, there was no significant language effect or interaction.

Further, morphological facilitation effect was measured by subtracting the semantic condition from the morphological condition in L1 and L2 Chinese, respectively. Chinese morphological facilitation effect (semantic minus morphological: 25.8 ± 33 ms) seems to be greater than English (−0.7 ± 72 ms) on RT (*p* = 0.06), while L1 Chinese and L2 English showed comparable ACC patterns (*p* = 0.91).

### fNIRS results

Two-way repeated-measures ANOVA (language and priming type) were conducted on beta values channel by channel. Three channels [Channel #1 (96.98% overlapping with Frontopolar area): *F*(1,29) = 6.9019, *p* = 0.0136, partial η^2^ = 0.1922; Channel #3 (96.23% overlapping with Frontopolar area): *F*(1,29) = 8.382909, *p* = 0.007128, partial η^2^ = 0.224244; Channel #19 (47.83% overlapping with Pre-Motor and Supplementary Motor Cortex): F(1,29) = 8.982393, *p* = 0.00554, partial η^2^ = 0.236488] showed significant main effect of language (*p*s < 0.05), such that hemodynamic responses significantly increased from English tasks to Chinese ones. Meanwhile, another three channels manifested a significant priming type effect, yet with distinct patterns [Channel #9 (92.08% overlapping with DLPFC): *F*(1, 29) = 4.941975, *p* = 0.034166, partial η^2^ = 0.145601. Channel #15 (50.5% overlapping with STG): *F*(1, 29) = 7.842814, *p* = 0.008987, partial η^2^ = 0.212872. Channel #16 (37.42% overlapping with STG and 31.13% with MTG): *F*(1,29) = 8.410107, *p* = 0.007046, partial η^2^ = 0.224808.]. Morphological priming elicited significantly greater brain activations than semantic priming in Channel #9 [dorsolateral prefrontal cortex (DLPFC)] (*p* < 0.05), while semantic priming involved more activations than morphological conditions in Channels #15 and #16 [superior temporal gyrus (STG) and middle temporal gyrus (MTG)] (*p*s < 0.01).

**Fig. 2:**
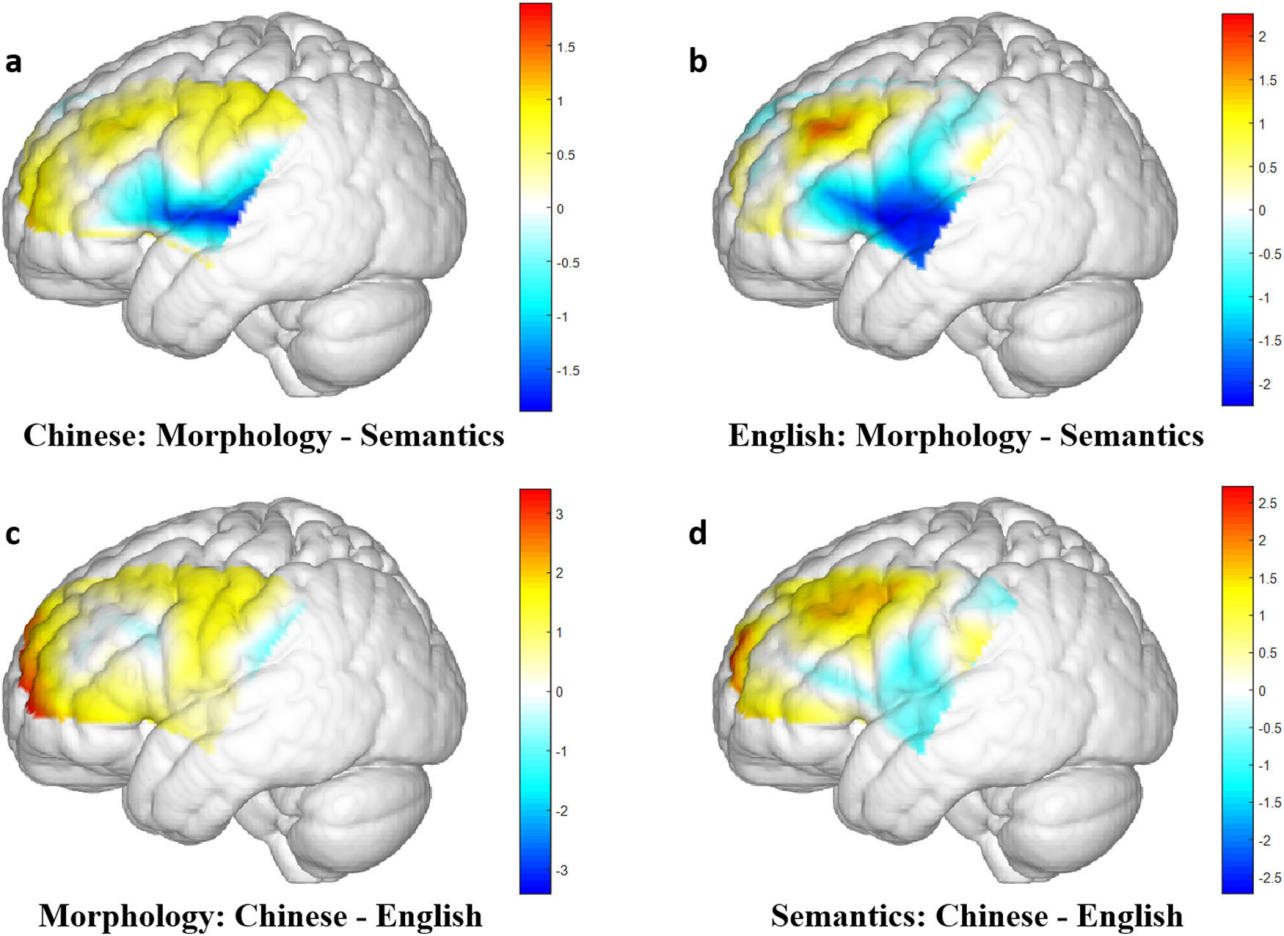
Brain activation difference in four pairwise comparisons. **a** Morphological vs. semantic processing in Chinese. **b** Morphological vs. semantic processing in English. **c** Chinese vs. English difference in morphological processing. **d** Chinese vs. English difference in semantic processing. Color bar denotes the *t* values between contrasts.

To further detect the morphological priming effect and cross-linguistic effect, four pairwise comparisons were conducted (Fig. 2) and significant results were summarized in Table 1. Specifically, Chinese semantic priming was more pronounced than morphological priming in STG and MTG (Channels #15 and 16). English morphological priming involved more activations in DLPFC (Channel #9), while English semantic priming employed more hemodynamic responses in STG and MTG (Channels #15 and 16). In terms of cross-linguistic morphological priming effect, Chinese morphology was more prominent in the frontopolar area (Channels #1, 3, 4) as compared to English. Likewise, Chinese semantic priming engaged more significant activation in frontopolar area (Channel #3) than English.

**Table 1:** Channels with significant results in pairwise comparisons.

Comparisons	Ch#	MNI (x y, z)	BA	Anatomical label (overlap)	*t*	unadjusted *p*	FDR adjusted *p*
Chinese: Morphology - Semantics	15	(−60, 10, 4)	22	Superior Temporal Gyrus (50.5%)	−2.7422	0.0103	0.0244
	16	(−70, −18, 3)	22	Superior Temporal Gyrus (37.42%)	−3.1488	0.0038	0.0151
			21	Middle Temporal gyrus (31.13%)			
English: Morphology - Semantics	9	(−47, 32, 37)	9	Dorsolateral Prefrontal Cortex (58.75%)	2.2577	0.0317	0.1267
			46	Dorsolateral Prefrontal Cortex (33.33%)			
	15	(−60, 10, 4)	22	Superior Temporal Gyrus (50.5%)	−2.6740	0.0122	0.0244
	16	(−70, −18, 3)	22	Superior Temporal Gyrus (37.42%)	−2.5166	0.0176	0.0353
			21	Middle Temporal Gyrus (31.13%)			
	17	(−48, −7, −16)	21	Middle Temporal Gyrus (95.71%)	−2.1077	0.0438	0.1753
Morphology: Chinese-English	1	(−38, 63, −1)	10	Frontopolar area (96.98%)	3.4094	0.0019	0.0077
	3	(−19, 65, 26)	10	Frontopolar area (96.23%)	2.1866	0.0370	0.0740
	4	(−8, 65, 32)	10	Frontopolar area (72.06%)	2.9279	0.0066	0.0263
Semantics: Chinese-English	3	(−19, 65, 26)	10	Frontopolar area (96.23%)	2.7185	0.0110	0.0438

Paired-sample *t*-test, two-sided.

*Ch#* fNIRS Channel number, *FDR* false discovery rate.

Additionally, we compared L1 Chinese and L2 English by using the brain activation difference between morphological priming and semantic priming. Only a marginal significant difference was identified from Channel #10 (80% overlapping with DLPFC), *t*(29) = 1.88, *p* = 0.07, where Chinese morphological effect showed enhanced activation than English.

### ERP results

**Fig. 3:**
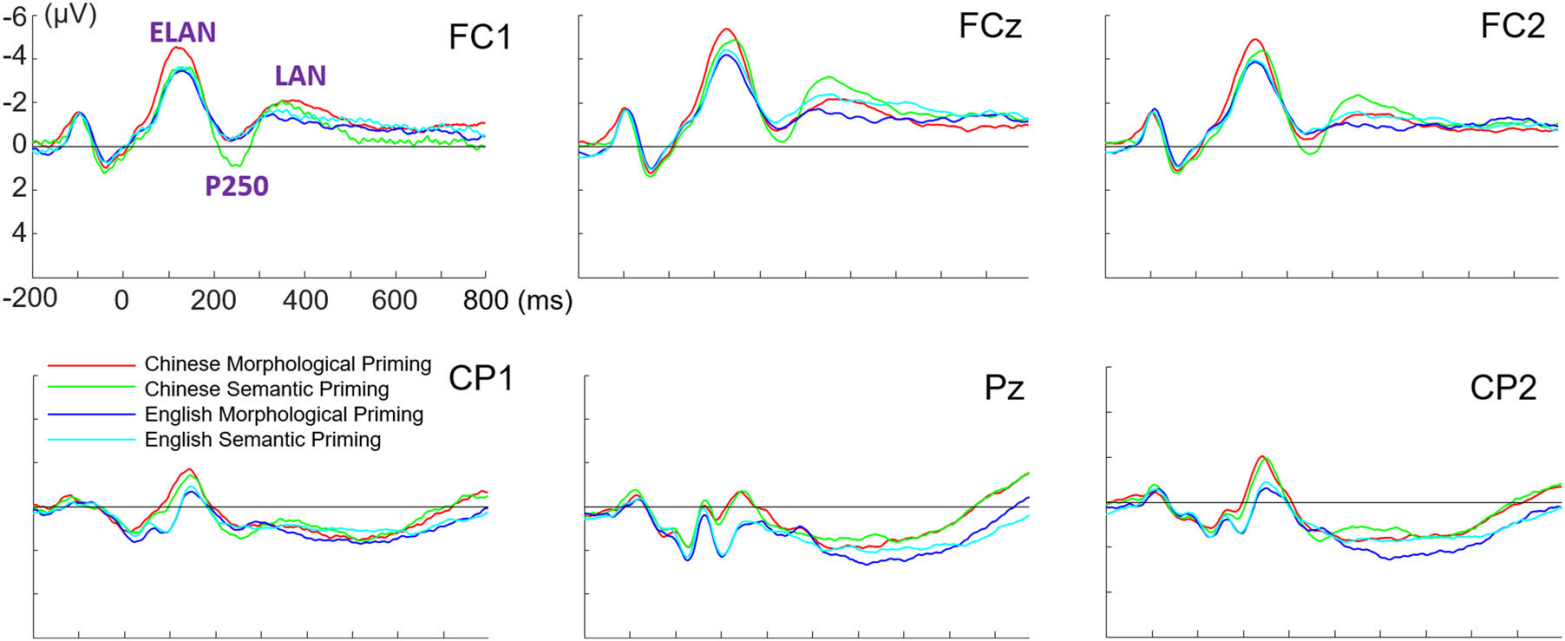
The grand-average ERP waveforms from anterior and posterior sites. ELAN, P250, and LAN effects were marked on representative electrodes.

As can be seen from the grand-average brainwaves (Fig. 3), ERPs showed an early negativity at the left anterior region (ELAN), followed by a positivity peaking around 200 ~ 250 ms after target word onset (P250), and a left negative deflection around 300 ~ 500 ms (LAN). To examine the effects of the three ERP components, three-way repeated measures ANOVA was performed with language (L1 Chinese vs. L2 English), priming type (morphological vs. semantic), laterality (left: AFF5h, FC1, CPP5h, CP1, P3; midline: FCz, Pz; right: FC2, FC6, P4, CP2, CP6), and region (anterior: AFF5h, FC1, FCz, FC2, FC6; posterior: CPP5h, CP1, P3, P4, CP2, CP6) as factors.

80 ~ 150 ms

**Fig. 4:**
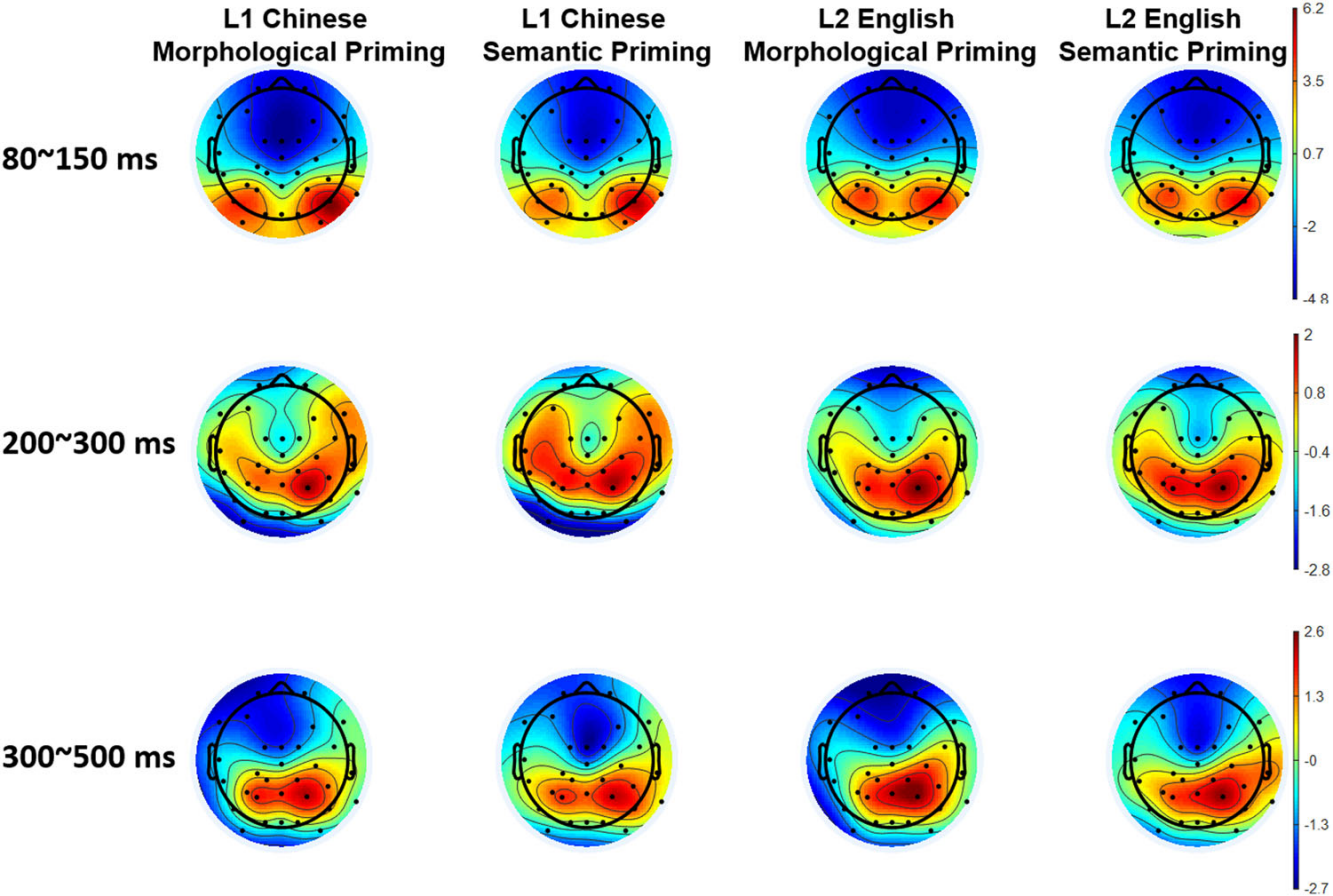
Averaged topographies within selected time windows. 80~150 ms: ELAN effect was identified mainly from the anterior sites. 200~300 ms: significant P250 was detected. 300~500 ms: LAN effect was prominent in the frontal cortex.

Based on the scalp distribution of the brain potentials from 80 to 150 ms (Fig. 4), we interpreted this pattern as an ELAN. There was a significant main effect of language, *F*(1,29) = 41.362, *p* < 0.001, partial η^2^ = 0.588, such that L1 Chinese (−1.2 ± 0.23 μV) elicited enhanced negativity than L2 English (−0.52 ± 0.21 μV). Meanwhile, the observed negativities were more pronounced in anterior regions (−3.28 ± 0.42 μV) as compared to posterior electrodes (1.57 ± 0.25 μV) (*p* < 0.001), and in midline than bilateral areas (*p*s < 0.001). ELAN was enhanced from the right hemisphere (−0.44 ± 0.2 μV) to the left one (−0.53 ± 0.26 μV), even though the difference did not reach a statistical difference. The three-way interaction between region, language, and priming type was marginal significant, *F*(1,29) = 3.849, *p* = 0.059, partial η^2^ = 0.117. Following analyses showed than Chinese morphological priming was associated with significant greater negativity in the anterior regions (−3.8 ± 0.51 μV) than Chinese semantic priming (−3.14 ± 0.44 μV) (*p* = 0.03), while this morphological priming effect on ELAN was absent in English tasks (*p* = 0.61).

We also compared the neural differences associated with behavioral morphological facilitation (morphological priming minus semantic priming) in light of a three-way ANOVA with language, laterality, and region as factors. The interaction between language and region showed a marginal significance (*p* = 0.059), such that Chinese morphological ELAN was greater than English.

200 ~ 250 ms

While previous studies identified a frontal P250 effect associated with semantic and structural priming^14,39^, the P250 component we identified was enhanced in centro-parietal cortex, as can be seen from Fig. 4. There was a significant region effect, *F*(1,29) = 10.631, *p* = 0.003, partial η^2^ = 0.263, such that posterior region engaged significantly greater positivity (1.14 ± 0.25 μV) than the anterior region (−0.53 ± 0.4 μV). The interaction between region and language was significant, *F*(1,29) = 4.997, *p* = 0.033, partial η^2^ = 0.147. Follow-up comparisons revealed that Chinese tasks involved enhanced negativity in anterior cortex, in relative to English tasks (*p* = 0.048). In addition, there was also a significant interaction between laterality and priming type [*F*(2,58) = 5.597, *p* = 0.011, partial η^2^ = 0.162], such that semantic priming elicited significantly greater positivity than morphological priming in the left hemisphere (*p* = 0.005). No other language- or priming-related main effect and interaction was identified. In addition, difference wave analysis (morphological priming minus semantic priming) on P250 showed no significant language effect or language-related interaction.

300 ~ 500 ms

Negativity was significantly greater in the frontal cortex (−1.37 ± 0.38 μV) than posterior regions (1.7 ± 0.24 μV), more enhanced in the left hemisphere (−0.08 ± 0.25 μV) than the right hemisphere (0.63 ± 0.21 μV) (*p*s < 0.05). We therefore interpretated this pattern as a LAN effect. Meanwhile, there was a significant interaction effect between region, laterality, and priming type, *F*(2,58) = 9.99, *p* = 0.002, partial η^2^ = 0.256. Simple main effect results showed that semantic priming (−2.33 ± 0.44 μV) involved greater negativity in the midline electrode of fronto-central cortex, as compared to morphological priming (−1.61 ± 0.53 μV) (*p* = 0.013). Additionally, difference wave analysis on LAN showed no significant language effect or language-related interaction. All tests in the current study were two-tailed.

## Discussion

In light of a derivational morphology, this study examined how and when cross-language similarity might modulate the neural resources employed by L1 and L2 morphological processing in Chinese-English adult bilinguals’ brain. Specifically, we aimed at identifying the shared and unique temporal signatures as well as brain bases of cross-language morphological processing strategies, irrespective of linguistic typological difference. The behavioral performance of the current study confirmed both reliable language effect and morphological priming facilitation. While L1 Chinese manifested shorter reaction time and higher accuracy rate, the Chinese-English bilinguals ranging from intermediate to advanced levels were engaged in both Chinese (ACC: 96.73%) and English tasks (ACC: 93.67%). In addition, as the test materials drew on a common morphology between L1 and L2, we postulate the results we obtained were not much contaminated by language proficiency.

Importantly, we identified a prominent facilitation effect of morphological priming relative to semantic priming. Yet, the following analysis further showed that this facilitation was primarily driven by L1 Chinese morphological effect, as there was no significant reaction time difference between English morphological and semantic priming. This pattern therefore suggested that the distinction between cross-language morphological sensitivity might take place at the sub-lexical level. The morphological facilitation effect which was observed in Chinese morphological priming yet absent in English could implicate that bilinguals employ a morphological parsing strategy only in their L1. While Gao, et al.^40^ revealed a whole-word priority (as manifested by whole-word repetition priming) in compound word representation as compared to morpheme priming, both whole-word and morpheme play important roles in mental lexicon. In addition, there is another possibility that L1 Chinese could primarily utilize a decomposition method for derivational words, as to date little psycholinguistic research touched upon the cognitive mechanisms of Chinese derivational words. Following ERP and fNIRS analyses would further examine whether and how L2 morphological processing resembles or differs from that of L1 at the neural level.

The brain activation patterns identified by fNIRS data revealed a core hub at the left prefrontal cortex for bilingual morphological processing. In light of the ANOVA results, morphological priming was associated with increased hemodynamic responses in DLPFC, as compared to semantic priming. Following pairwise comparisons revealed that this main effect was driven by L2 English, while there was no reliable difference between Chinese morphological priming and semantic priming. In contrast, pure semantic association between prime and target manifested greater brain activation in the left temporal cortex including STG and MTG, as compared to that of morphological relationship. The current results therefore present a neural dissociation between morphological and semantic relationship encoding across L1 and L2 in bilingual individuals. First, we found that the left frontal cortex implicates a language-general brain region for morphology in bilingual processing. This finding corroborates existing studies^25,26^ employing differing task demands and language modalities. Second, the left temporal cortex is also crucial for morphology vs. semantics differentiation, where semantic processing might overweigh morphological parsing. Previous MEG study^28^ found that reading morphologically complex Chines words (e.g., compound) would elicit larger brain activities in the left anterior and posterior temporal cortex relative to monomorphemic control. In addition, Ip, et al.^27^ detected significant activation in superior and middle temporal cortex in a Chinese word construction task, which was linked to automatic phonological and semantic analysis. As both studies abovementioned did not rule out semantic contamination in morphological tasks, we postulate that the brain patterns observed in the left temporal regions could be largely driven by semantic analysis. As such, activation in the left prefrontal cortex might be a robust indicator for the commonality of Chinese-English bilingual morphological processing.

On the other hand, the current fNIRS results shed light upon a L1 vs. L2 difference in the left frontal cortex. Compared to L2 English, L1 Chinese generated greater brain activation in the frontopolar area (BA 10) for both morphological and semantic priming conditions. This cross-linguistic brain difference pattern is in line with the contrast results between Chinese and English monolingual readers’ word reading^41,42^, except that we did not identify a language difference in MTG. The discrepancy could be attributed to task natures, as Tan and his colleges’ studies focused on the characteristics of Chinese addressed phonology, while our study concentrated on the morphological and semantic aspects of lexical processing. Nevertheless, the current results contrasted with those from Chinese-English bilingual children. In particular, Ip, et al.^36^ found that bilingual children manifested greater activation in the left frontal region in L2 English relative to L1 Chinese and associated this pattern with a language-specific neural pattern for morphological processing. They ascribed the inconsistency between their study and monolingual study to two possible reasons. First, it might result from the differing morphological constraints from L1 and L2, as they used Chinese compound morphology and English derivational morphology for test materials. Second, bilingual children might be more proficient in English than in Chinese at the time of test, since all participant children were fully exposed to English by age 4 and had at least 4 years of bilingual exposure before the experiment. By contrast, the current study recruited primarily late bilinguals, whose English proficiency would outperform Chinese by no means. In addition, the morphological relationship under investigation is present for both languages. By using this cross-language similarity, the current study did not find dissociable brain regions which were responsible for L1 Chinese and L2 English morphological processing, respectively. Instead, L1 and L2 showed quantitative difference in the left frontal cortex. It suggests that this region is associated with native-ness and competence in morphology. More importantly, this pattern implicates that bilinguals could transfer their L1 abilities to L2 performance, as IFG has been recognized as a crucial hub for Chinese word reading^26,41,42^. This finding therefore provided further evidence to the unified competition model that language structures shared in L1 and L2 might rely on the common L1 neurocognitive resources during bilingual development.

Interestingly, current ERP results also identified shard and distinct electrophysiological patterns for bilingual morphological processing. First, both L1 and L2 brainwaves manifested an ELAN component, which is a temporal signature in the brain for automatic syntactic processing and initial structure building^43,44^. It indicates that bilinguals could employ similar processing strategy for both L1 and L2 at the early stage of lexical processing. In addition, we found that morphological priming condition was associated with greater ELAN than semantic priming when bilingual readers were recognizing words in L1 Chinese. By contrast, there was no statistical difference between the two priming conditions in L2 English. As such, early structural sensitivity in L1 and L2 shows difference in degree, yet not in kind. Especially, ELAN has been taken as an indicator for native-ness in second language morpho-syntactic processing and acquisition^45–47^. There is high chance that bilinguals could demonstrate identical ELAN patterns for L1 and L2 morphological processing, conditional on adequate bilingual exposure and proficiency.

In the time window of 200 ~ 300 ms, we identified a P250 effect, such that semantic priming elicited enhanced positivity than morphological priming in the left hemisphere. P250 has been associated with the early semantic processing accompanied with short SOAs in both German^39^ and Chinese^14,15^. In particular, word pairs with semantic associations generated greater P250 than those with a morphological relationship, which was taken as an indicator of automated structural priming and morphological parsing^15^. The current results replicated the previous findings and extended the morphological priming P250 effect to Chinese derivational words. Importantly, this pattern applies to both L1 and L2 within bilinguals’ brain. P250 results suggest that both L1 and L2 might engage an affix-stripping process, thus making both languages manifest a morphological priming facilitation over semantic relationship, while our behavioral and ELAN results showed such an effect only for L1 Chinese. Therefore, it could be implicated that P250 work as a shared indicator of structural priming and morphological sensitivity in bilingual word reading.

Following P250, we identified a negativity pronounced at left anterior region. We interpret this pattern as a LAN effect based on its scalp distribution. LAN was associated with morpho-syntactic error integration^11,12,48^ in sentence processing and structural problem resolution in word reading^9,13^. While the current study detected a morphological priming facilitation by LAN pattern, there was no significant interaction between language and priming type. As such, the observed LAN could be an extension of previous structural parsing, which implicates a language-general component within bilingual processing. Alternatively, there is another possible explanation for the negativity observed between 300 ~ 500 ms. We could also interpret this pattern as an anterior N400, which constitutes a classic language component associated with lexico-semantic processing^49,50^. In either manner, the late negativity implicates a conscious and controlled evaluation on the morphological constraints for both L1 and L2.

In conclusion, the current study provided behavioral and neural evidence on the shared and distinct pattern of L1 and L2 morphological processing in adult bilinguals’ brain by using simultaneous behavioral, electrophysiological, and hemodynamic responses. Our fNIRS results revealed that while morphological and semantic priming could be dissociable in the left frontal and temporal regions across languages, L1 Chinese engaged enhanced activation in the left prefrontal cortex for morphological parsing relative to L2 English. Meanwhile, L1 and L2 shared both automatic morphological parsing and structural priming at the sub-lexical level, as well as top-down processing at the lexical level. In particular, ELAN effects manifested the cross-language morphological processing as a difference in degree, not in kind. Collectively, the current results support a unified competition model for bilingual development. Especially for language structures which are shared between L1 and L2, bilinguals primarily employ a common processing strategy, thus transferring L1 resources into L2 use. However, even though the bilingual adults were engaged in both Chinese and English tasks, so far we cannot completely rule out the proficiency confounding effect on current results. To eliminate this confound, future studies could include monolinguals as controls, so as to better evaluate the neural correlates of language native-ness in bilingual processing by comparing L2 patterns with the monolinguals of that language. Meanwhile, language proficiency could be manipulated as a factor, such that the modulation of proficiency on the shared and different patterns between L1 and L2 could be well addressed. In addition, the current study analyzed EEG and fNIRS data in parallel, so as to provide the time-course and cortical activation information of morphological processing, respectively. Future studies could integrate EEG-fNIRS data to obtain interesting brain patterns.

## Methods

### Participants

Thirty Mandarin-Chinese native speakers (15 males; mean age 22.2 years old, SD = 3.2 years, age range: 18–30) were recruited from the University of Macau as paid participants. All participants were righthanded as assessed by Edinburgh Handedness Inventory^51^, with normal or corrected-to-normal vision and no reported brain disease or mental disorders. They started to learn English as a foreign language at 7.4 ± 2.2 years old and have not stayed in an English-speaking country for more than six months. Prior to the formal experiment, participants were instructed to complete the LexTALE^52^ test online to examine their English vocabulary proficiency. The average score is 57.21/100 (SD = 6.74), roughly indicating a medium level on average (the cutoff of medium level is 60%). Research protocol and materials were approved by the Institutional Review Board of University of Macau. All participants signed on the informed consent form before the formal test. All measurements were taken from distinct samples.

### Stimuli

Sixty Chinese derivational words of high frequency (averaged log frequency: 1.65) accessed from SUBTLEX-CH dataset^53^ served as target words in Chinese condition. Those derivational words engaged 6 (semi-)prefixes (e.g., 准-/zhun3/, someone-to-be) and 40 (semi-)suffixes (e.g., -家/jia1/, -expert), which were retrieved from a summary of Chinese affixes^54^. In a constitute priming scenario, they were primed by their corresponding word roots. For instance, the prime of 音乐家(/yin1 yue4 jia1/, musician) is 音乐 (/yin1 yue4/, music). Sixty Chinese compound words were used in semantic priming as control condition, whose meanings were closely related to their primes. Primes were retrieved from Small World of Words (smallworldofwords.org/zh/project) project, which provides a dataset of Chinese word association. Yet, there is not any morphological, orthographic, or phonological relationship between prime and target.

Likewise, 60 English words with suffixes (e.g., -er, -or, -ness, -ment) and their frequency information were retrieved from SUBTLEX-US dataset^55^. Sixty semantically related word pairs were selected from University of South Florida Free Association Norms dataset (Nelson, McEvoy, & Schreiber, 1998) and thus used as semantic controls.

Both Chinese and English words were incorporated into a semantic judgment task, where participants were asked to judge whether the target word’s meaning is people-related or not. In each condition (Chinese morphological priming, Chinese semantic priming, English morphological priming, English semantic priming), half of the target words were directly related to people semantically (e.g., runner, wife), while the other half were not. All target words were validated by seven college students with various English proficiency levels, whose offline judgments on people-relatedness were perfectly consistent. Another 8 volunteers on campus were invited to rate their familiarity towards 120 targets words from 1 to 9, whose results revealed a high familiarity (>8.2) with both Chinese and English words. Meanwhile, 9 raters evaluated the semantic association between primes and corresponding targets across all conditions, indicating a close relatedness. Additionally, the semantic distances between prime and target within each word pair were computed by using the pre-trained BERT (Bidirectional Encoder Representations from Transformers) model^56^, where a bigger value would indicate a longer semantic distance. Information of word log frequency, word length, number of strokes, familiarity, subjective semantic association, and semantic distance was provided in Table 2. To verify participants’ familiarity with those materials, we verbally checked how familiar they were with those words and whether there were words they did not know after their formal experiments. Their results validated the prior familiarity ratings.

**Table 2:** Lexical characteristics and examples of primes and targets across conditions.

Language	Priming Type	Material	Prime				Target					Semantic Association	Semantic Distance
			Example	Frequency	Word Length	Stroke Number	Example	Frequency	Word Length	Stroke Number	Familiarity		
Chinese (L1)	Morphological priming	People-related	歌/ge1/, song	2.9 (1.1)	1.7 (0.5)	13.9 (6.8)	歌手/ge1 shou3/, singer	1.9 (0.8)	2.7 (0.5)	20.2 (7)	8.3 (0.5)	7.0 (0.6)	4.0 (1.3)
		Non people-related	木/mu4/, wood	2.9 (0.8)	1.7 (0.5)	13 (5.3)	木头/mu4 tou2/, wood	1.4 (0.9)	2.7 (0.5)	20.7 (6.4)	8.2 (0.6)	6.8 (0.9)	3.8 (1.5)
	Semantic priming	People-related	大海/da4 hai3/, ocean	2.9 (0.9)	1.8 (0.4)	13 (3.7)	海盗/hai3 dao4/, pirate	2.6 (1.2)	2.2 (0.4)	17.6 (6.3)	8.3 (0.5)	6.8 (0.7)	5.2 (2.7)
		Non people-related	晴/qing2/, sunny	3 (1.1)	1.7 (0.4)	14.9 (4.5)	蓝天/lan2 tian1/, blue sky	2.9 (1.2)	2.2 (0.4)	16.6 (4.7)	8.4 (0.5)	6.7 (0.7)	5.2 (2.8)
English (L2)	Morphological priming	People-related	teach	3.3 (0.6)	5.8 (1.8)	/	teacher	2.5 (0.7)	8.1 (1.6)	/	8.6 (0.5)	7.1 (0.5)	5.4 (1.1)
		Non people-related	happy	3.5 (0.6)	5.4 (1.6)	/	happiness	2.7 (0.7)	8.3 (1.9)	/	8.6 (0.7)	7.2 (0.6)	4.8 (1.4)
	Semantic priming	People-related	hospital	3.6 (0.5)	5 (1.5)	/	nurse	3.4 (0.5)	5.7 (2)	/	8.6 (0.6)	6.5 (0.7)	4.8 (0.9)
		Non people-related	fly	3.9 (0.6)	5.3 (1.5)	/	bird	3.6 (0.6)	6.1 (1.7)	/	8.7 (0.5)	6.5 (0.7)	4.6 (1.3)

### Procedure

**Fig. 5:**
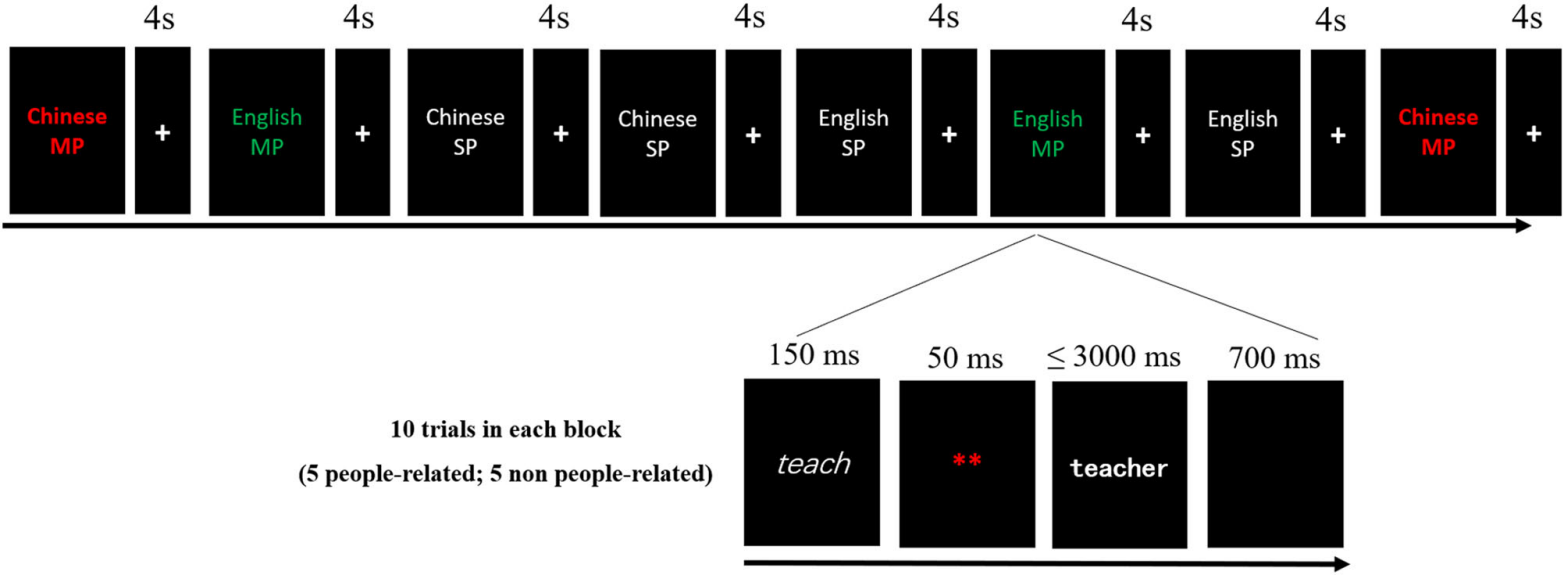
Schematic procedure of experimental procedure. Participants need to decide whether the target is semantically people-related or not. A stimulus-onset asynchrony (SOA) of 200 ms between prime and target is used. MP morphological priming, SP semantic priming.

Participants performed a semantic judgment task in a block design. There were three runs with 8 blocks in each run (2 blocks for each condition within one run). There were 10 trials in each block with 5 people-related and 5 non-people-related targets presented in a randomized order. Every two consecutive blocks were spaced by a fixation at the screen center lasting for 4 s. Each trial started from a prime with a duration of 150 ms, which was then followed by 50-ms mask consisting of two red asterisks. After the mask disappeared, the target word would show up. Participants were instructed to decide whether the target word is people-related or not by pressing the corresponding buttons in the keyboard. Target words would be present until a response was made or after 3 s, which was then replaced by a blank of 700 ms. Experimental procedure was visualized in Fig. 5.

### EEG and fNIRS recordings and analysis

EEG and fNIRS signals were acquired at the same time when participants were performing lexical decision tasks (Fig. 1a). We recorded fNIRS data by using NIRScout system, which measures optical signals with the wavelengths of 760 nm and 850 nm and a sampling rate of 7.81 Hz. Eight sources and eight detectors constitute 22 fNIRS channels, covering the frontal and temporal cortex of the left hemisphere. The distance between a source and its corresponding detectors was kept at 3 cm. In particular, the MNI coordinates of the 22 channels were retrieved from the international 10/20 system and then submitted to NIRS_SPM software^57^ to obtain their Brodmann area (BA), anatomical label, and percentage of overlap. For offline analysis, fNIRS data were segmented in the format of blocks and categorized into the four conditions (Chinese morphological priming, Chinese semantic priming, English morphological priming, and English semantic priming), which were then filtered with a band-pass of 0.01–0.2 Hz by using nirsLAB software. In addition, discontinuities and spike artifacts were removed by the algorithms provided by the software. Next, hemodynamic states were computed by using the Beer-Lambert law. Then, oxygenated hemoglobin (HbO) data were submitted to General Liner Model (GLM) estimation in light of a hrf function. As a result, beta values of four conditions from each participant were obtained.

We recorded EEG data by using Brainvision’s acti-Champ system with 32 active electrodes with a sampling rate of 500 Hz and a reference of the left mastoid. Electrode impedances were kept below 25 kΩ during signal acquisition. The obtained EEG data were pre-processed offline by using EEGLAB in MATLAB environment. First, data were re-referenced to grand average and filtered with a bandpass of 0.01–30 Hz. Then, epochs were extracted ranging from 200 ms before target onset to 800 ms afterward. Independent Component Analysis (ICA) was performed to identify and remove eye blinks and line noise. Next, trials with voltages exceeding ±100 μV were treated as artifacts and discarded. If a single channel showed extensive artifacts even after the artifact correction, it would be interpolated by averaging the voltages of spheric electrodes.

### Supplementary information

reporting-summary

## Data Availability

All stimuli, data, and scripts used in the analyses can be found at https://osf.io/29e3j/.

## Code availability

The R scripts we used for data analyses are available at https://osf.io/29e3j/.
